# Phenolic Composition and Antioxidant Activity of *Malus domestica* Leaves

**DOI:** 10.1155/2014/306217

**Published:** 2014-09-11

**Authors:** Mindaugas Liaudanskas, Pranas Viškelis, Raimondas Raudonis, Darius Kviklys, Norbertas Uselis, Valdimaras Janulis

**Affiliations:** ^1^Department of Pharmacognosy, Faculty of Pharmacy, Lithuanian University of Health Sciences, Eivenių Street 4, 50161 Kaunas, Lithuania; ^2^Institute of Horticulture, Lithuanian Research Centre for Agriculture and Forestry, Kauno Street 30, Babtai, 54333 Kaunas, Lithuania

## Abstract

The aim of this study was to determine the composition and content of phenolic compounds in the ethanol extracts of apple leaves and to evaluate the antioxidant activity of these extracts. The total phenolic content was determined spectrophotometrically, as well as the total flavonoid content in the ethanol extracts of apple leaves and the antioxidant activity of these extracts, by the ABTS, DPPH, and FRAP assays. The highest amount of phenolic compounds and flavonoids as well as the highest antioxidant activity was determined in the ethanol extracts obtained from the apple leaves of the cv. Aldas. The analysis by the HPLC method revealed that phloridzin was a predominant component in the ethanol extracts of the apple leaves of all cultivars investigated. The following quercetin glycosides were identified and quantified in the ethanol extracts of apple leaves: hyperoside, isoquercitrin, avicularin, rutin, and quercitrin. Quercitrin was the major compound among quercetin glycosides.

## 1. Introduction

Studies on the chemical biodiversity of plants are recognized as being relevant and are carried out with the aim of enriching the assortment of raw medicinal plant materials and to evaluate their potential application to the demands of practical medicine. A search for plants accumulating phenolic compounds, which have recently been considered as an object of many scientific studies, is especially promising. It is of importance to assess the composition and content of phenolic compounds in plant vegetative organs, to determine the patterns of their accumulation and identify new, promising sources of plant phenolic compounds.

The domestic apple (*Malus domestica* Borkh.) is one of the most widely cultivated fruit trees. Although the chemical composition of apples has been extensively investigated [1, 2], we have failed to find any data on the composition and content of phenolic compounds in the leaves of different apple cultivars grown in Lithuania. Comprehensive data of scientific research on the variation in the composition and content of phenolic compounds would allow conducting purposeful studies leading to the usage of the raw material obtained from apple leaves as a potential source of phenolic compounds in practical medicine. Biologically active compounds could lead to production of dietary supplements and cosmetic preparations enriched in phenolic compounds found in apple leaves. Small-scale studies on the chemical composition of leaves have been published, where phloretin glycosides, phenolic acids, catechins, and some quercetin glycosides were identified as the main phenolic compounds [3–5]. Other studies on the composition and content of phenolic compounds in apple leaves were conducted in relation to* Venturia inaequalis*-caused infections in the vegetative organs of apple trees. Phenolic compounds accumulated in fruit plants play an important role in the plant defense mechanism against different fungal diseases and different stresses [6–9].

Phenolic compounds, acting as natural antioxidants, scavenge free radicals and inhibit their production, stimulate the synthesis of antioxidant enzymes, and thus prevent oxidative stress resulting in damage to the structural molecules of the body [10–12]. The antioxidant activity of phenolic compounds is associated with other biological properties of these compounds, such as anti-inflammatory, antimicrobial, anticancer, cardiovascular system-improving, and other activities [13–15]. Apple leaves accumulate high amounts of phloridzin [16, 17], which exhibit antidiabetic activity [18]. Therefore, plant raw materials accumulating this compound can be potentially useful for the prevention of diabetes mellitus.

Therefore, the aim of this study was to determine the composition and content of phenolic compounds in the ethanol extracts of apple leaves harvested from the cultivars Aldas, Auksis, Ligol, and Lodel grown under Lithuanian climatic conditions, to assess the variation of phenolic compounds and to compare their antioxidant activity in the apple leaf samples obtained from different cultivars.

## 2. Materials and Methods

### 2.1. Plant Material

The following apple cultivars were included into this study: Auksis (early winter cv., bred in Lithuania) and Ligol (winter cv., bred in Poland) that are two main cultivars in commercial apple orchards as well as Aldas (early winter cv., bred in Lithuania) and Lodel (winter cv., bred in Poland), two cultivars resistant to apple scab (*Venturia inaequalis*). Cultivars Aldas and Lodel are recommended for organic orchards. The apple trees were grown in the experimental orchard of the Institute of Horticulture, Lithuanian Research Centre for Agriculture and Forestry, Babtai, Lithuania (55°60′N, 23°48′E). Aldas: block 4, row 16, trees 5–8; Auksis: block 2, row 3, trees 25–28; Ligol: block 2, row 4, trees 21–24; Lodel: block 4, row 5, trees 12–15. The altitude of Babtai town is 57 m above sea level. Average annual precipitation is 630 mm, and average sum of active temperatures is (>10°C)–2300°. Temperatures during the experiment year were close to long term average. The summer of 2012 was characterised by more rainfall in April, June, and July. Trees were trained as slender spindle. Pest and disease management was carried out according to the rules of the integrated plant protection. The experimental orchard was not irrigated. Tree fertilization was performed according to soil and leaf analysis. Nitrogen was applied before flowering at the rate of 80 kg/ha, and potassium was applied after harvest at the rate of 90 kg/ha. Soil conditions of the experimental orchard were as follows: clay loam, pH: 7.3, organic matter: 2.8%, P_2_O_5_: 255 mg/kg, and K_2_O: 230 mg/kg. Apple leaves were harvested from 10-year-old apple trees in 2012. The study sample comprised 20 healthy, fully developed leaves collected from different places of each apple tree of each cultivar. Apple leaves were lyophilized with a ZIRBUS sublimator 3 × 4 × 5/20 (ZIRBUS technology, Bad Grund, Germany) at a pressure of 0.01 mbar (condenser temperature, –85°C). The lyophilized apple leaves were ground to a fine powder by using a Retsch 200 mill (Haan, Germany). Loss on drying before analysis was determined by the method of the European Pharmacopoeia [19]. Data were recalculated for absolute dry lyophilizate weight.

### 2.2. Chemicals

All the solvents, reagents, and standards used were of analytical grade. Acetonitrile and acetic acid were obtained from Sigma-Aldrich GmbH (Buchs, Switzerland) and ethanol from Stumbras AB (Kaunas, Lithuania). Hyperoside, rutin, quercitrin, phloridzin, phloretin, caffeic acid, and chlorogenic acid standards were purchased from Extrasynthese (Genay, France), (+)-catechin and (–)-epicatechin from Fluka (Buchs, Switzerland) and avicularin and isoquercitrin from Chromadex (Santa Ana, USA). 1,1-Diphenyl-2-picrylhydrazyl (DPPH^•^) radical, 2,2′-azino-bis(3-ethylbenzothiazoline-6-sulphonic acid) (ABTS), potassium persulfate, sodium acetate trihydrate, iron (III) chloride hexahydrate, and 2,4,6-tripyridyl-s-triazine (TPTZ) were obtained from Sigma-Aldrich (Steinheim, Germany). Folin-Ciocalteu reagent, gallic acid monohydrate, sodium carbonate, aluminum chloride hexahydrate, and hexamethylenetetramine were purchased from Sigma-Aldrich GmbH (Buchs, Switzerland). Deionized water, produced by the Crystal E high-performance liquid chromatography (HPLC, Adrona SIA, Riga, Latvia) water purification system, was used.

### 2.3. Extraction

An amount of 0.25 g of lyophilized apple leaf powder (exact weight) was weighed, added to 10 mL of ethanol (70%, v/v), and extracted in a Sonorex Digital 10 P ultrasonic bath (Bandelin Electronic GmbH & Co. KG, Berlin, Germany) for 40 minutes at 60°C. The extract obtained was centrifuged for 7 minutes at 6000 rpm with a Hermle Z206A centrifuge (Denville Scientific Inc., USA). The extract was collected and filtered through a membrane filter with a pore size of 0.22 *μ*m (Carl Roth GmbH, Karlsruhe, Germany).

### 2.4. Spectrophotometric Studies

#### 2.4.1. Determination of Total Phenolic and Flavonoid Content

All the spectrophotometric measurements were carried out with a Genesys-10 UV/Vis spectrophotometer (Thermo Spectronic, Rochester, USA). The total phenolic content (mg GAE/g DW) in the ethanol extracts of apple leaves was determined by the Folin-Ciocalteu method [20]. The total amount of flavonoids in the ethanol extracts of apple leaves was determined using the described methodology [21], calculated from a rutin calibration curve, and expressed as mg/g rutin equivalent (RE) per gram of absolutely dry weight (DW) (mg RE/g DW).

#### 2.4.2. Determination of Antioxidant Activity

*(1) DPPH* ^•^ *Free Radical Scavenging Assay*. The DPPH^•^ free radical scavenging activity was determined using the method proposed by Brand-Williams et al. [22]. DPPH^•^ solution in 96.3% v/v ethanol (3 mL, 6 × 10^−5^ M) was mixed with 10 *μ*L of the ethanol extract of apple leaves. A decrease in absorbance was determined at a wavelength of 515 nm after keeping the samples for 30 minutes in the dark.

*(2) ABTS* ^•*+*^ *Radical Cation Decolorization Assay.* An ABTS^•+^ radical cation decolorization assay was applied according to the methodology described by Re et al. [23]. A volume of 3 mL of ABTS^•+^ solution (absorbance 0.800 ± 0.02) was mixed with 10 *μ*L of the ethanol extract of apple leaves. A decrease in absorbance was measured at a wavelength of 734 nm after keeping the samples for 30 minutes in the dark.

*(3) FRAP Assay*. The ferric reducing antioxidant power (FRAP) assay was carried out as described by Benzie and Strain [24]. The working FRAP solution included TPTZ (0.01 M dissolved in 0.04 M HCl), FeCl_3_·6H_2_O (0.02 M in water), and acetate buffer (0.3 M, pH 3.6) at the ratio of 1 : 1 : 10. A volume of 3 mL of a freshly prepared FRAP reagent was mixed with 10 *μ*L of the apple leaf extract. An increase in absorbance was recorded after 30 minutes at a wavelength of 593 nm.

*(4) Calculation of Antioxidant Activity of the Ethanol Extract of Apple Leaves.* The antioxidant activity of extracts was calculated from Trolox calibration curve and expressed as *μ*mol Trolox equivalent (TE) per gram of absolutely dry weight (DW). TE was calculated according to the formula: TE = *c* × *V*/*m* (*μ*mol/g); *c*: the concentration of Trolox established from the calibration curve (in *μ*M); *V*: the volume of leaf extract (in L); *m*: the weight (precise) of lyophilized leaf powder (in g).

### 2.5. High-Performance Liquid Chromatography

Qualitative and quantitative analysis of phenolic compounds was performed according to the* previously validated* and described high-performance liquid chromatography (HPLC) method [25]. A Waters 2695 chromatograph equipped with a Waters 2998 photodiode array (PDA) detector (Waters, Milford, USA) was used for HPLC analysis. Chromatographic separations were carried out by using a YMC-Pack ODS-A (5 *μ*m, C18, 250 × 4.6 mm i.d.) column equipped with a YMC-Triart (5 *μ*m, C18, 10 × 3.0 mm i.d.) precolumn (YMC Europe GmbH, Dinslaken, Germany). The column was operated at a constant temperature of 25°C. The volume of the extract being investigated was 10 *μ*L. The flow rate was 1 mL/min, and gradient elution was used. The mobile phase consisted of 2% (v/v) acetic acid in water (solvent A) and 100% (v/v) acetonitrile (solvent B). The following conditions of elution were applied: 0–30 minutes, 3%–15% B; 30–45 minutes, 15%–25% B; 45–50 minutes, 25%–50% B; and 50–55 minutes, 50%–95% B.

The identification of the chromatographic peaks was achieved by comparing the retention times and spectral characteristics (*λ* = 200–600 nm) of the eluting peaks with those of reference compounds. The compounds identified were confirmed by spiking the sample with the standard compound and monitoring the changes in the peak shape and spectral characteristics. For quantitative analysis, a calibration curve was obtained by injection of known concentrations of different standard compounds. Dihydrochalcones and catechins were quantified at 280 nm, phenolic acids at 320 nm, and flavonols at 360 nm.

### 2.6. Statistical Data Processing Methods

All the experiments were carried out in triplicate. Means and standard deviations were calculated with the SPSS 20.0 software (Chicago, USA). A single factor analysis of variance (ANOVA) along with the post hoc Tukey test was employed for statistical analysis. The Kolmogorov-Smirnov test was applied to examine the normality of distribution. To verify the hypothesis about the equality of variances, Levene's test was employed. The correlation was evaluated by Pearson analysis. Differences at *P* < 0.05 were considered to be significant.

## 3. Results and Discussion

### 3.1. Determination of Total Phenolic and Flavonoid Contents

In order to determine the patterns of the accumulation of biologically active compounds in plants, it is important to identify their composition and content in separate plant organs. The secondary metabolites of plant metabolism, phenolic compounds, have been detected in apple leaves [3, 16]; therefore, this study aimed at determining the composition and content of phenolic compounds in plants and at determining the patterns of their variation and accumulation.

In this study, the total amount of phenolic compounds in the ethanol extracts of apple leaves varied from 98.81 ± 1.51 mg GAE/g DW (cv. Auksis) to 163.35 ± 4.36 mg GAE/g DW (cv. Aldas) (Table 1). In a study by Iqbal et al. [26], the total phenolic content in the ethanol extracts of apple leaves was 157.06 mg GAE/g DW; meanwhile one Slovenian study reported a lower total phenolic content in apple leaves, ranging from 80 mg GAE/g DW to 115 mg GAE/g DW [16]. Both scab-resistant cultivars, Aldas and Lodel, accumulated significantly higher amounts of total phenolics and total flavonoids. Such differences among the cultivars susceptible and resistant to apple scab have been established in other studies as well [27].

Studies investigating the total phenolic content in the leaf samples of other plants belonging to the* Rosaceae* family have been conducted as well. Methanol extracts from the leaves of various* Sorbus* species were found to have lower total amounts of phenolics ranging from 60.6 to 90.9 mg GAE/g DW [28]. Hua et al. [6], who investigated the samples of pear leaves, reported a lower total phenolic content (68.1–83.3 mg GAE/g DW).

The biological effects of many plant raw materials depend on flavonoids; therefore, studies on the variation in their content are important and relevant. This study determined the total flavonoids content in the ethanol extracts of apple leaves, which ranged from 21.59 ± 0.52 mg RE/g DW (cv. Auksis) to 45.02 ± 0.90 mg RE/g DW (cv. Aldas) (Table 1). Iqbal et al., who investigated the ethanol extracts of apple leaves, reported a higher total flavonoids content, reaching 121.86 mg RE/g DW [26]. In contrast to that, the ethanol extracts of hawthorn leaves were found to have a lower total flavonoids content (9.5–13.0 mg RE/g DW) [21].

The data on the patterns of variation in the total phenolic and flavonoid contents of apple leaves are scarce. Therefore, this study provides new knowledge about total phenolics and flavonoids content in the apple leaves of the cultivars grown under Lithuanian climatic conditions, allows the comparison of the results obtained with those of other studies, and is valuable to carrying out a search for promising, biologically active substance-accumulating plant raw materials.

### 3.2. Measurements of Antioxidant Activity in Extracts

After studying the total phenolics and flavonoids content of apple leaves harvested from different cultivars grown under Lithuanian climatic conditions, it is important to examine and assess the antioxidant activity in the extracts of apple leaves. The results obtained during studies will be useful for the selection of apple cultivars in order to provide a consumer with products rich in antioxidants, will be useful for the assessment and standardization of quality of plant raw materials and their products, and will allow predicting an antioxidant effect of apple leaves* in vivo*.

**Figure 1:**
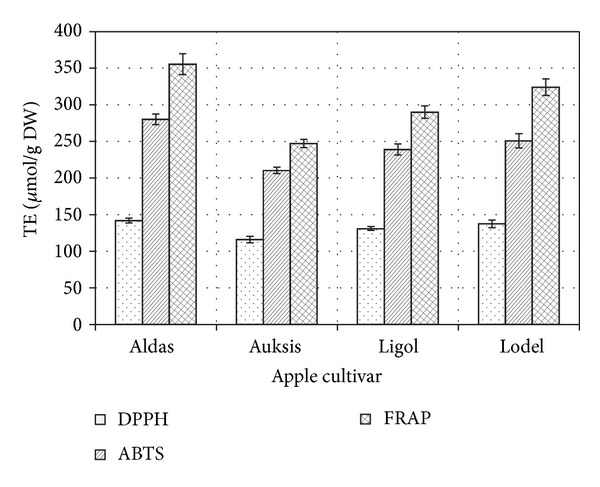
The antioxidant activity of ethanol extracts obtained from apple leaves and determined by using DPPH, ABTS, and FRAP assays. Values are means and errors bars indicate standard deviations (*n* = 3).

Herbal extracts are multicomponent matrices with antioxidant activity determined by the set of different mechanism reactions, so antioxidant effect cannot be adequately tested using only one method [29, 30]. For these reasons, it is recommended to use at least two different methods for determination of antioxidant activity in herbal extracts [31]. In order to thoroughly evaluate the antioxidant activity of the ethanol extracts of apple leaves, different antioxidant capacity assays (DPPH, ABTS, and FRAP) were employed. The* in vitro* antioxidant effect of the investigated extracts was evaluated by the DPPH and ABTS assays as a capability of DPPH^•^ and ABTS^•+^, compounds possessing an antiradical activity, to scavenge free radicals [22, 23] and by the FRAP assay, as a capability of antioxidants to reduce Fe(III) to Fe(II) [24]. The results of* in vitro* antioxidant activity determined in the ethanol extracts of apple leaves (cultivars Aldas, Auksis, Ligol, and Lodel) are summarized in Figure 1.

It has been previously reported that antioxidant capacity determined by* in vitro* assays differs [32–34]. TE values, obtained from samples of apple leaf extracts antioxidant activity studies, according to the used methods, can be arranged in the following order: DPPH < ABTS < FRAP (Figure 1). This pattern could be explained by differences of applied antioxidant activity determination methods. FRAP assay is a method for measuring total reducing power of electron donating substances, whilst ABTS and DPPH assays are methods for measuring the ability of antioxidant molecules to scavenge ABTS^•+^ and DPPH^•^ free radicals, respectively [35, 36]. ABTS and DPPH methods have several differences from one another, which causes their TE values to be different. Some authors indicate that TE or VCE values determined by DPPH method are lower than those determined by ABTS method [37–40] probably because the DPPH method has more limitations. ABTS radical cation (ABTS^•+^) is soluble in water and organic solvents, which allows determining antiradical activity of the hydrophilic and lipophilic compounds [30, 41]. DPPH^•^ radicals are only soluble in organic solvents, which limits the evaluation of antioxidant activity of hydrophilic antioxidants [36, 42]. Wang et al. found that some of the compounds, which have ABTS^•+^ scavenging activity, may not show DPPH^•^ scavenging activity [43]. Arts et al. reported that some products of ABTS^•+^ scavenging reaction may have a considerable contribution to the antioxidant capacity and can continually react with ABTS^•+^ [44].

The analysis of the antioxidant activity in apple leaves revealed differences in the antioxidant activity among the ethanol extracts of the apple leaves of the cultivars investigated (Figure 1). The ethanol extracts obtained from the apple leaves of the cv. Aldas showed the highest TE values. They reached 141.95, 280.23, and 355.54 *μ*moL/g DW for the DPPH, ABTS, and FRAP assays, respectively. To our knowledge, no studies on the antioxidant activity of apple leaves have been carried out so far. Therefore, the findings from this study on antioxidant activity were compared with those obtained in studies on other plants belonging to the* Rosaceae* family. The methanol extracts obtained from the leaves of different* Sorbus* species showed a higher antioxidant activity; that is, the TE values were higher than those determined in the present study: 344.0–628.3 *μ*moL/g DW by the DPPH assay, 262.5–467.3 *μ*moL/g DW by the ABTS assay, and 861.6–1650.4 *μ*moL/g DW by the FRAP assay [28]. These results could be explained by the fact that apple leaves accumulate high amounts of phloridzin, belonging to the dihydrochalcone class [3, 16], which possesses a lower antioxidant activity than the phenolic compounds belonging to other classes [4, 45].

In order to determine the relationship between the antioxidant activities of the ethanol extracts of apple leaves assessed by the DPPH, ABTS, and FRAP assays and total phenolic as well as flavonoid contents in these extracts, the correlation analysis was carried out. There was a strong positive correlation between total phenolic as well as flavonoid contents and the antioxidant activity assessed by all the methods (*r* = 0.84–0.98, *P* < 0.05). The strongest correlation was found between the antioxidant activity of the ethanol extracts of apple leaves determined by the FRAP method and total flavonoid content (*r* = 0.96, *P* < 0.05) as well as total phenolic content (*r* = 0.98, *P* < 0.05) in these extracts. Other studies published earlier also reported strong correlative relationships between phenolic as well as flavonoid contents and the antioxidant activity of plant extracts evaluated by different assays [46–48].

### 3.3. Identification and Quantification of Phenolic Compounds by HPLC

**Figure 2:**
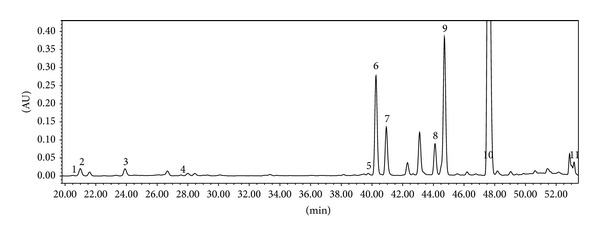
Chromatogram of ethanol extract of apple leaf sample (cv. Aldas) investigated (*λ* = 320 nm). Numbers indicate the peaks of analytes: 1: (+)-catechin, 2: chlorogenic acid, 3: caffeic acid, 4: (–)-epicatechin, 5: rutin, 6: hyperoside, 7: isoquercitrin, 8: avicularin, 9: quercitrin, 10: phloridzin, and 11: phloretin.

The phenolic compounds of various groups, (+)-catechin, chlorogenic acid, caffeic acid, (–)-epicatechin, rutin, hyperoside, isoquercitrin, avicularin, quercitrin, phloretin, and phloridzin, in the analyzed ethanol extracts obtained from the apple leaves of the cultivars Aldas, Auksis, Ligol, and Lodel were identified by the HPLC method. The values of resolution (Rs > 2) were achieved in all the samples of extracts. The applied HPLC method allowed for an effective separation of quercetin glycosides: the separation of rutin and hyperoside in ethanol extracts of lyophilized apple leaf samples with the resolution of 2.63; the separation of hyperoside and isoquercitrin with the resolution of 3.28; and the separation of avicularin and quercitrin with the resolution of 2.94. The chromatograms of ethanol extracts of apple leaf samples obtained from all the cultivars investigated are identical regarding the number of analytes and retention time. The example of chromatogram of the ethanol extract of the apple leaf sample (cv. Aldas) is shown in Figure 2.

The apple leaves of the cv. Aldas contained the highest total amount of the quercetin glycosides identified and quantified. It was 1.7 times higher than their lowest amount found in the apple leaves of the cv. Ligol (Table 2). Quercitrin was a predominant component among all the quercetin glycosides identified and quantified in the ethanol extracts of apple leaves. The apple leaves of the cv. Aldas had the highest amount of quercitrin. The results obtained are in agreement with literature data that quercitrin is one of the main flavonols found in apple leaves [5, 49]. The results of this study are in line with those reported by the Slovenian study where the quercitrin content in the apple leaves collected in the Maribor region ranged from 6.3 mg/g to 10.8 mg/g (cv. Golden Delicious) and from 3.7 mg/g to 8.5 mg/g (cv. Jonagold) [49]. The factors such as a geographical region and climatic-meteorological and cultivation conditions could influence these quantitative differences.

Rutin was the minor component among all the quercetin glycosides quantified in all ethanol extracts of the apple leaf samples analyzed. The results obtained are confirmed by previously published data on the variation in the composition and content of quercetin glycosides in apple leaves [5]. The highest amount of rutin was detected in the apple leaves of the cv. Auksis (Table 2). However, the Slovenian study reported contrary findings on the flavonol content in apple leaves. The authors of that study demonstrated that rutin along with quercitrin was one of the major compounds in apple leaves [49].

When comparing quercetin glycoside content and composition variation in apple fruits and leaves grown in Lithuanian climatic conditions, it was found that apple leaves have higher amounts of these compounds. For example, cv. Aldas, cv. Auksis, and cv. Ligol apple fruits had hyperoside amounts from 0.05 mg/g (cv. Auksis) to 0.19 mg/g (cv. Aldas) [25] which constitutes only 0.9–2.1% hyperoside content found in leaves of the same apple cultivars. Similar quantitative differences between apple fruit and leaf grown in Lithuania are also typical in other quercetin group compounds.

All the quercetin glycosides identified and quantified in the ethanol extracts of apple leaf samples can be ranked in the following ascending order by their content: rutin < isoquercitrin< avicularin < hyperoside < quercitrin. This order was characteristic of the apple leaves of the cultivars Auksis, Ligol, and Lodel. In the apple leaves of the cv. Aldas, the amount of isoquercitrin was higher than that of avicularin.

Phloridzin and phloretin, which belong to the dihydrochalcone class, were identified in the ethanol extracts of apple leaf samples. Phloridzin was the major phenolic compound in the apple leaves. It accounted for 76.9% to 84.2% of all phenolic compounds identified and quantified in the extracts of apple leaves by the HPLC method. The results obtained confirm literature data that phloridzin is a predominant component of phenolic compounds in apple leaves [16, 17]. Determined phloretin levels were significantly lower. They make up 1.0–1.8% of all identified and quantified phenolic compounds. This could be interpreted by phloretin and phloridzin molecules structural differences. Phloridzin is phloretin glycoside, which has glucose linked at hydroxy group in molecule's 2′ position. Gosch et al. researched dihydrochalcone synthesis processes in apple leaves and found that phloridzin is formed from phloretin by glycosylation reaction, affected by enzyme dihydrochalcone 2′-O-glucosyltransferase [17]. Sugar molecule link to the aglycone influences the physical-chemical properties of flavonoid. Commonly flavonoid glycosides have lower antioxidant activity and are more hydrophilic than their respective aglycones [50, 51]. These characteristics relate to the fact that the flavonoid aglycone has more free hydroxy groups than their corresponding glycosides, which have one or several hydroxy groups with bonded sugar [52, 53]. Rezk et al. indicates that formation of phloridzin by glycosylation of hydroxy group at phloretin molecule's 2′ position reduces its antioxidant activity 18 times compared to phloretin [54]. Other researchers who conducted comparative studies of phloretin and phloridzin anti-inflammatory effects and their influence on lipid oxidation report similar patterns. Phloretin has significantly stronger anti-inflammatory [55] and lipid oxidation inhibitory effect [56] than phloridzin. The flavonoid aglycone reactivity explains why glycosides are the most common flavonoid in plants [57, 58]. This pattern is also characteristic for dihydrochalcone group compounds in apple leaves—glycoside phloridzin levels are significantly higher than its aglycone phloretin levels [3, 59].

Phloridzin has a wide spectrum of biological effects, inhibits the growth of cancer cells [60], improves memory [61, 62], and is useful in the bone fracture prevention [63, 64]. One of the most important and potentially valuable phloridzin biological effects is antidiabetic activity [18, 65]. Main phloridzin pharmacological mechanism of action, leading to its antidiabetic effect, is to produce renal glycosuria and block intestinal glucose absorption through inhibition of sodium-glucose symporters in kidneys and small intestine [66]. The principle uses of phloridzin are associated with its ability to reduce plasma glucose, without changing insulin levels [67, 68]. Phloridzin ability to reverse glucotoxicity and reduce blood glucose levels without increasing body weight determines its benefits in prophylaxis and treatment of type 2 diabetes [69–71]. Phloridzin reduces body weight by blocking the absorption and resorption of glucose [72, 73], and the weight loss is one of the most important type 2 diabetes prevention methods [74, 75]. Phloridzin consumption to reduce glucose concentration in blood plasma does not cause body fluid loss and hypoglycemia risk [66, 76]. For the reasons stated above, it is purposeful to investigate plant materials and extracts that accumulate phloridzin. We believe it is a promising research in developing medicines and food supplements for body weight reduction in the prevention of diabetes. Our assumption is supported by other scientist's research, which offers to use apple leaves to enrich phenolic compounds composition with phloridzin in apple juice [77].

The apple leaves of the cv. Ligol contained the highest concentration of phloridzin. The identified amounts of phloretin were lower than those of phloridzin (Table 3). Such trends in the content of compounds belonging to this dihydrochalcone class have been demonstrated by other studies as well [3]. In contrast to the leaves of apple cultivars grown in Lithuania, phloridzin was not the dominant compound in apple fruits. Determined phloridzin levels were 0.06–0.14 mg/g [25], and it was only 3.9–5.5% of identified and quantified phenolic compounds, tested in varieties cv. Aldas, cv. Auksis, and cv. Ligol. There was also a distinction of composition of phenolic compounds between the apple fruit and the apple leaf samples. Phloretin was identified only in apple leaf samples but not in apple fruit samples [25, 78].

The highest total amount of phenolic acids identified and quantified by the HPLC method (1.61 ± 0.07 mg/g) was found in the apple leaves of the cv. Auksis (Table 3). The ethanol extracts of apple leaves contained caffeic acid; its amount was lower than that of chlorogenic acid. The levels of chlorogenic acid were similar or slightly higher than those reported by the earlier studies [49, 79]. Fruit and leaf composition and content of apples cultivated in Lithuanian climatic conditions differed. In cv. Aldas, cv. Auksis, and cv. Ligol apples, chlorogenic acid was the predominant compound; determined amounts (0.69–2.23 mg/g) were 54.8–69.6% of all identified and quantified phenolic compounds [25]. In apple leaf extracts, phloridzin was dominant and chlorogenic acid was only 0.3–1.0% of all identified phenolic compounds. Composition differences were also determined; apple leaves contained caffeic acid, while apple fruits did not.

The chemical composition of catechins (monomeric flavan-3-ols) in ethanol extracts obtained from the apple leaves of the cultivars Aldas, Auksis, Ligol, and Lodel was studied. The highest and lowest total amounts of the catechins identified were found in the apple leaves of the cultivars Ligol and Lodel, respectively (0.85 ± 0.04 mg/g versus 0.56 ± 0.02 mg/g) (Table 3). The amounts of (–)-epicatechin in the extracts studied were higher than those of (+)-catechin. Similar amounts of (–)-epicatechin were reported by other authors [3]. (+)-Catechin (0.05–0.15 mg/g) and (–)-epicatechin (0.24–0.45 mg/g) levels determined in apple fruits of Lithuanian cultivated cv. Aldas, cv. Auksis, and cv. Ligol were similar to those determined in apple leaves. Composition differences were also determined—procyanidins (oligomeric flavan-3-ols) group compounds; procyanidin B1 and procyanidin B2 were identified in apple fruits [25, 78], while in apple leaves they were not.

The HPLC analysis of ethanol extracts obtained from apple leaves revealed that phloridzin was the major compound in the samples investigated, and its amounts were considerably higher than those of other phenolic compounds. Quercitrin was a predominant component among quercetin glycosides. The results of the HPLC analysis show that apple leaves are a valuable, natural source of dihydrochalcones and quercetin glycosides. This encourages further research on this plant as a raw material for use in pharmacy.

## 4. Conclusions

In conclusion, the results of this study will provide new knowledge about the composition and content of phenolic compounds in apple leaves and the antioxidant activity of their extracts, which will give a wide range of possibilities to employ these plants as the source of phenolic compounds. The highest total amounts of phenolic compounds and flavonoids were determined in the apple leaves of the cv. Aldas (163.35 ± 4.36 mg GAE/g DW and 45.02 ± 0.90 mg RE/g DW, resp.). Phloridzin was the major compound in the ethanol extracts of apple leaves of all the cultivars investigated. The apple leaves of the cv. Ligol had the highest amount of phloridzin (114.43 ± 4.72 mg/g DW). Quercitrin was the predominant component among the quercetin glycosides identified and quantified in ethanol extracts, and its amount in the apple leaves of different cultivars ranged from 7.77 to 13.36 mg/g DW.

The preliminary* in vitro* experiments examining the antioxidant activity of apple leaf extracts by the ABTS, DPPH, and FRAP assays have shown that these extracts possess a strong antioxidant activity, which positively correlated with the total phenolic and flavonoid contents (*r* = 0.84–0.98, *P* < 0.05). The ethanol extracts obtained from the apple leaves of the cv. Aldas showed the highest TE values: 141.95 *μ*mol/g DW by the DPPH assay, 280.23 *μ*mol/g DW by the ABTS assay, and 355.54 *μ*mol/g DW by the FRAP assay.

The results reported in this study prompt further research on the chemical composition and biological effect of apple leaves by evaluating the antioxidant activity of individual phenolic compounds* in vitro* and* in vivo* and confirm a potential of these plants as a raw material in medical practice as well as the development and production of dietary supplements and cosmetic preparations rich in biologically active compounds.

## Figures and Tables

**Table 1:** The total amounts of phenolic compounds (TP) and flavonoids (TFd) in the ethanol extracts of apple leaves of different cultivars.

cv.	TP (mg GAE/g DW)^a^	TFd (mg RE/g DW)^a^
Aldas	163.35 ± 4.36	45.02 ± 0.90
Auksis	98.81 ± 1.51	21.59 ± 0.52
Ligol	107.93 ± 2.94	26.97 ± 0.63
Lodel	159.86 ± 4.02	39.64 ± 1.31
	*P* < 0.05^b^	*P* < 0.05^b^

^a^Values are means ± standard deviations (*n* = 3).

^b^By ANOVA test.

**Table 2:** Content of quercetin glycosides in ethanol extracts obtained from the apple leaves of cultivars grown in Lithuania.

Compound	Content of quercetin glycosides, mg/g (expressed for absolute dry weight)^a^			
	Aldas	Auksis	Ligol	Lodel
Hyperoside	8.95 ± 0.35^a^	5.67 ± 0.20^b^	4.59 ± 0.17^c^	7.03 ± 0.20^d^
Isoquercitrin	3.48 ± 0.12^a^	2.79 ± 0.11^b^	1.84 ± 0.07^c^	2.40 ± 0.08^d^
Rutin	0.33 ± 0.01^a^	0.75 ± 0.03^b^	0.67 ± 0.02^c^	0.54 ± 0.02^d^
Avicularin	2.48 ± 0.10^a^	2.82 ± 0.10^b^	2.09 ± 0.07^c^	2.51 ± 0.08^d^
Quercitrin	13.36 ± 0.51^a^	10.29 ± 0.48^b^	7.77 ± 0.27^c^	12.31 ± 0.59^d^

^a^Values are means ± standard deviations (*n* = 3). The different letters indicate significant differences between the values (*P* < 0.05).

**Table 3:** Content of dihydrochalcones, phenolic acids, and catechins in ethanol extracts obtained from the apple leaves of cultivars grown in Lithuania.

Compound	Content of dihydrochalcones, phenolic acids, and catechins, mg/g (expressed for absolute dry weight)^a^			
	Aldas	Auksis	Ligol	Lodel
Phloridzin	106.01 ± 4.23^a^	108.9 ± 4.32^b^	114.43 ± 4.72^c^	109.51 ± 4.62^d^
Phloretin	1.81 ± 0.07^a^	1.52 ± 0.06^b^	2.40 ± 0.09^c^	1.40 ± 0.06^d^
Chlorogenic acid	0.48 ± 0.02^a^	1.38 ± 0.06^b^	1.12 ± 0.05^c^	0.86 ± 0.03^d^
Caffeic acid	0.26 ± 0.02^a^	0.23 ± 0.03^b^	0.15 ± 0.01^c^	0.14 ± 0.01^d^
(+)-Catechin	0.05 ± 0.01^a^	0.27 ± 0.02^b^	0.09 ± 0.01^c^	0.17 ± 0.01^d^
(−)-Epicatechin	0.72 ± 0.02^a^	0.38 ± 0.02^b^	0.76 ± 0.02^c^	0.39 ± 0.01^d^

^a^Values are means ± standard deviations (*n* = 3). The different letters indicate significant differences between the values (*P* < 0.05).

## Abbreviations

ANOVA: A single factor analysis of variance
ABTS: 2,2′-azino-bis(3-ethylbenzothiazoline-6-sulphonic acid)
cv.: Cultivar
DPPH: 2,2-diphenyl-1-picrylhydrazyl
DW: Dry weight
FRAP: Ferric reducing antioxidant power
GAE: Gallic acid equivalent
HPLC: High-performance liquid chromatography
Rs: Resolution
TE: Trolox equivalent
TFd: Total amount of flavonoids
TP: Total amount of phenolic compounds
TPTZ: 2,4,6-tripyridyl-s-triazine
VCE: Vitamin C equivalent.
